# Maternal mortality in adolescents and young adults: temporal trend and correlation with prenatal care coverage in the state of Bahia, Brazil, 2000-2020

**DOI:** 10.1590/S2237-96222023000200022

**Published:** 2023-09-18

**Authors:** Lorena Ramalho Galvão, Maria Conceição Oliveira Costa, Silvana Granado Nogueira da Gama, Magali Teresópolis Reis Amaral, Djanilson Barbosa dos Santos, Naysa Farias Barros, Wanessa Oliveira Rosario

**Affiliations:** 1 Universidade Estadual de Feira de Santana, Programa de Pós-Graduação em Saúde Coletiva, Feira de Santana, BA, Brazil; 2 Fundação Oswaldo Cruz, Escola Nacional de Saúde Pública Sergio Arouca, Rio de Janeiro, RJ, Brazil; 3 Universidade Federal do Recôncavo da Bahia, Centro de Ciências da Saúde, Santo Antônio de Jesus, BA, Brazil

**Keywords:** Time-Series Studies, Epidemiological Studies, Prenatal Care, Maternal Mortality, Adolescent, Young Adult., Estudios de Series Temporales, Estudios Epidemiológicos, Atención Prenatal, Mortalidad Materna, Adolescente, Adulta Joven, Estudos de Séries Temporais, Estudos Epidemiológicos, Cuidado Pré-Natal, Mortalidade Materna, Adolescente, Adulto Jovem

## Abstract

**Objective:**

to analyze the temporal trend of maternal mortality and correlate it with prenatal care coverage among adolescents and young adults, state of Bahia, Brazil, 2000-2020.

**Methods:**

this was an ecological time-series study and correlation between maternal deaths and prenatal care visits in 10-19 and 20-24 age groups, using the Mortality Information System; the trend analysis was performed by means of Prais-Winsten regression, according to race/skin color, timing and causes of death; and Spearman coefficient was used for correlation.

**Results:**

in the study period, 418 deaths among adolescents and 574 among young adults were recorded; maternal mortality ratio was 59.7 and 63.2 deaths/100,000 live births, with a significant decreasing trend (-2.2% and -2.9% respectively); it could be seen an inverse correlation between a higher number of prenatal care visits and maternal mortality in the age groups.

**Conclusion:**

maternal mortality showed a decreasing trend in the study period, but with high proportions of death; there was a significant correlation between prenatal care coverage and maternal mortality among adolescents and young adults.

## Study contributions

**Main results:**

From 2000 to 2020, maternal mortality among adolescents and young adults showed a decreasing trend in the state of Bahia. It could be seen an inverse and significant correlation between the highest number of prenatal care visits and maternal mortality in the studied groups.

**Implications for services:**

The study suggests the importance of quality obstetric care during prenatal, childbirth, and postpartum period for reducing maternal mortality among adolescents and young adults, especially from preventable causes.

**Perspectives:**

Improving the records of causes of death on information systems, enhancing obstetric care, and investing in sexual and reproductive health policies aimed at adolescents may contribute to the reduction of maternal deaths.

## RESUMEN

**Objetivo:**

analizar la tendencia temporal de la mortalidad materna y correlacionarla con la cobertura prenatal, entre adolescentes y adultas jóvenes, en el estado de Bahía, período 2000-2020.

**Métodos:**

estudio ecológico de series temporales y correlación entre muertes maternas y atención prenatal, en grupos de edad de 10-19 y 20-24, utilizando el Sistema de Información de Mortalidad. Analizó la tendencia mediante regresión de Prais-Winsten, según raza/color de piel, causas y hora de muerte; para correlación, el coeficiente de Spearman.

**Resultados:**

en el período se registraron 418 muertes de adolescentes y 574 de adultas jóvenes; razón de mortalidad materna de 59,7 y 63,2 muertes/100.000 nacidos vivos, con importante tendencia a la baja (-2,2%; -2,9%, respectivamente). Se observó correlación inversa entre el mayor número de consultas y la mortalidad materna en grupos.

**Conclusión:**

la mortalidad materna mostró tendencia descendente en el período, pero con altas proporciones de muertes. Hubo una correlación significativa entre la cobertura prenatal y la mortalidad materna, adolescente y adulta joven.

## RESUMO

**Objetivo:**

analisar a tendência temporal da mortalidade materna e correlacioná-la com a cobertura pré-natal entre adolescentes e adultas jovens, Bahia, Brasil, 2000-2020.

**Métodos:**

estudo ecológico de série temporal e correlação entre óbitos maternos e consulta de pré-natal aos 10-19 e 20-24 anos, utilizando-se o Sistema de Informações sobre Mortalidade; a tendência foi analisada pela regressão de Prais-Winsten, segundo raça/cor da pele, causas e momento do óbito; para a correlação, utilizou-se o coeficiente de Spearman.

**Resultados:**

no período, foram registrados 418 óbitos de adolescentes e 574 de adultas jovens; razão de mortalidade materna de 59,7 e 63,2 óbitos/100 mil nascidos vivos, com tendência decrescente significante (-2,2% e -2,9% respectivamente); observou-se correlação inversa entre maior número de consultas e mortalidade materna, nos grupos etários.

**Conclusão:**

a mortalidade materna apresentou tendência de redução no período, porém com altas proporções de óbito; houve correlação significante entre cobertura pré-natal e mortalidade materna, entre adolescentes e adultas jovens.

## INTRODUCTION

Maternal mortality due to complications related to pregnancy, childbirth and postpartum period is still high worldwide. In 2020, about 287,000 maternal deaths were reported in different contexts and countries, although only 1% occurred in developed countries.^1^ Taking into consideration that the majority of these deaths occur from preventable causes, maternal mortality is a sensitive indicator of health conditions and quality of life of a population, especially because of its close relationship with inadequate access to prenatal and childbirth care services.^2^

In Brazil, despite advances in prenatal care and important investments made by the Ministry of Health, the maternal mortality ratio (MMR) remains high, and disproportionate to the country’s level of economic development, taking into consideration that the MMR increased between 2019 and 2020, ranging from 58 to 75 deaths per 100,000 live births (LB), respectively, in that biennium.^3^ These parameters indicate that the country has not achieved the reduction in maternal mortality established in the Sustainable Development Goals (SDGs) yet.^4^

According to data from the Health Department of the state of Bahia (SESAB), in the period from 2000 to 2020, 3,136 maternal deaths were recorded in all age groups, resulting in an overall MMR of 69.0 deaths per 100,000 LB, which was higher in 2020: 81.1 maternal deaths/100,000 LB. Maternal deaths among adolescents (10 to 19 years old) accounted for 13.3% of the total; and among young adults (20 to 24 years old), it was 18.3%.^5^ ^),(^ ^6^

The main determinants of maternal mortality are the precarious prenatal and childbirth care conditions, and the need for pregnant women to seek childbirth care, in addition to comorbidities and low level of education.^7^ ^),(^ ^8^ It is also worth mentioning the problems arising from non-spontaneous abortion, a procedure not legally permitted in the country, except under specific conditions, in addition to excessive medicalization and indication for surgical interventions (cesarean sections), which are often unnecessary.^4^

With regard to prenatal care, the Ministry of Health establishes criteria for adequate follow-up: early initiation (first prenatal care visit up to the 12^th^ week of pregnancy); a minimum of six prenatal care visits; laboratory tests; tetanus vaccination; educational activities; pregnancy risk classification; and access to the referral center for outpatient and/or hospital care in high-risk pregnancies.^9^ ^),(^ ^10^ These criteria are fundamental. However, the Live Birth Information System (Sistema de Informações sobre Nascidos Vivos - SINASC), does not include all this information, especially for the analysis of aggregate data, given that it only provides the number of prenatal care visits.^11^

In the field of reproductive health, adolescent pregnancy has been a permanent issue on government health agendas for about four decades, and has been extensively discussed by professionals and researchers from different areas of knowledge. Official data attest that approximately 14.0% of births in the country in 2020 were to adolescent mothers, with higher rates in the poorest regions (North, 21.4%; and Northeast, 17.0%).^11^

In the least developed regions in the country, pregnant adolescents and adolescent mothers have low socioeconomic status, in addition to late initiation of prenatal care and inadequate access to childbirth care. The presence of one or more of these factors can contribute to complications during pregnancy, delivery and birth, leading to an impact on the pregnancy outcome and vitality of the pregnant woman, puerperal woman and newborn.^12^ ^),(^ ^13^

Young adult women, despite having lower biological risk, face socioeconomic vulnerability similar to that of adolescents due to labor market instability, low education, marital instability. These factors compromise their health status.^7^ ^),(^ ^14^

In this context, the present study aimed to analyze the temporal trend of maternal mortality and correlate it with prenatal care coverage among adolescents and young adults living in the state of Bahia, Brazil, from 2000 to 2020.

## METHODS

This was an ecological, time-series study aimed at analyzing maternal deaths among adolescents and young adults living in the state of Bahia, reported on the Mortality Information System (SIM), made available by the Brazilian National Health System Information Technology Department (Departamento de Informática do Sistema Único de Saúde - DATASUS) of the Ministry of Health, between 2000 and 2020.

The state of Bahia, located in the Northeast region of Brazil, has an estimated population of 15,666,521 inhabitants, with women of reproductive age (10 to 49 years) accounting for 31.2% of this total.^15^ In 2021, there were 185,459 births in the state, of which about 15% were to adolescent mothers and 24% to young adult women.^11^

Maternal age groups were defined according to the World Health Organization (WHO) criteria: adolescents (10-19 years old) and young adult women (20-24 years old). Maternal deaths were estimated based upon Chapter XV of the International Statistical Classification of Diseases and Related Health Problems, 10^th^ Revision (ICD-10) -, Pregnancy, childbirth and puerperium, in addition to maternal deaths classified in other chapters.^16^

The variables were analyzed in an aggregated manner, according to the year when the maternal death occurred (2000 to 2020), the age group (in full years: 10 to 19; and 20 to 24), race/skin color (White; Black; Mixed-race), types of obstetric causes (direct; indirect; unspecified), groups of maternal death causes and timing of death (during pregnancy, childbirth or abortion; or up to 42 days postpartum).

The direct and indirect causes of maternal death were defined according to the WHO classification, ICD-Maternal Mortality (ICD-MM),^16^ where codes from Chapter XV of ICD-10 were grouped as shown in Box 1.

Maternal mortality and birth data were extracted from the SIM and the SINASC in October 2022, using the publicly available TABNET tabulator in the DATASUS website. After tabulation, the data were exported to a spreadsheet.

**Box 1:** Groups of causes of maternal mortality and their respective codes, according to the International Statistical Classification of Diseases and Related Health Problems - 10^th^ Revision (ICD-10) - Maternal Mortality

Groups of causes	ICD-10^th^ codes
1 - Pregnancy with abortive outcome	O00-O07
2 - Hypertensive disorders in pregnancy, childbirth and the puerperium	O11-O16
3 - Obstetric hemorrhage	O20; O43-O46; O67; O71.0; O71.1, O71.3, O71.4, O71.7; O72
4 - Infections during pregnancy	O23; O41.1; O75.3; O85-O86; O91
5 - Other obstetric complications	O21.1, O21.2; O22; O24.4; O26.6, O26.9; O30-O36^b^; O40^b^; O41.0, O41.8, O41.9^b^; O42^b^; O60-061^b^; O62; 063-066^b^; O71.2, O71.5, O71.6, O71.8, O71.9; O73; O75.0-O75.2, O75.4-O75.9; O87.1, O87.3, O87.9; O88; O90
6 - Unanticipated complications (related to anesthesia)	O29; O74; O89
7 - Non-obstetric complications	O10; O24.0, O24.2, O24.3, O24.9; O98 (inclui B20-24) e O99
8 - Unspecified cause	O95

Source: adapted from the World Health Organization.^15^

a) ICD-10 = International Statistical Classification of Diseases and Related Health Problems - 10^th^ Revision -; b) Unlikely causes of maternal death, included in the group of other obstetric complications.

The coverage of prenatal care visits was calculated by dividing the number of visits (categorized variable: none; 1 to 6; 7 or more), available on SINASC, by the total number of live births in the same maternal age group and year, multiplied by 100. Additional criteria for prenatal care quality were not included because they are not available in TABNET/DATASUS.^11^

The cause-specific maternal mortality ratio (CSMMR) for each age group (adolescents and young adults) was calculated per year studied. In order to calculate the CSMMR, we used maternal deaths classified in Chapter XV of the ICD-10 [with the exception of codes O96 and O97: late maternal death, from any obstetric cause, occurring more than 42 days but less than one year after delivery (code 096); and maternal death from sequelae of direct obstetric cause, occurring one year or more after delivery (code 097)]. In the denominator, we used the total number of live births in the two age groups studied, extracted from SINASC, multiplied by 100,000. The CSMMR was also calculated according to race/skin color, types and groups of timing and causes of maternal death.

Regarding race/skin color, deaths among Asian and Indigenous mothers were excluded from the analyses due to their low frequency in the study period (among adolescents, Indigenous, n = 2, and Asian, n = 0; and among young adults, Indigenous, n = 1, and Asian, n = 3). Similarly, group 6 related to maternal death causes (unanticipated complications related to anesthesia) (adolescents, n = 2; and young adults, n = 3) was excluded.

Correction factors for maternal deaths were used in order to obtain more accurate estimates of CSMMR, based on recommendations in the literature.^17^ For the state of Bahia, the factor corresponding to the Northeast region was used (1.76 for the period 2000-2007; and 1.17 for 2008-2020).^17^ ^),(^ ^18^

Initially, a descriptive and exploratory analysis of the CSMMR was performed, calculating the mean and standard deviation. Then, the t-test was used to compare pair means for the variable at the time of death. Analysis of variance (ANOVA) and Tukey test were used to compare pairwise means for the variables “race/skin color” and “types of causes of death”. In all analyses, comparisons were made for each age group (adolescents and young adults), taking into consideration a significance level of 5%.

The correlation between the CSMMR and the proportion of prenatal care coverage was analyzed by calculating the Spearman’s Rank correlation coefficient. The temporal trend of maternal mortality was analyzed by means of Prais-Winsten regression model, where the ordinate axis (Y) represents the CSMMR values, and the abscissa axis (X) represents the period (2000-2020).^19^ Years without records of maternal deaths were excluded from the analysis.

The annual percentage change (APC) and its respective 95% confidence intervals (95%CI) was quantified for the period studied (2000-2020). Trends were considered increasing (p-value ≤ 0.05; positive regression coefficient), decreasing (p-value ≤ 0.05; negative regression coefficient) or stable (p-value > 0.05). Inferential analyses were performed using the Stata statistical software (version 14.0).

Since the study was based on secondary and anonymous publicly available data from SIM and SINASC, it was not necessary to submit the study project to a Research Ethics Committee (REC).

## RESULTS

**Figure 1:**
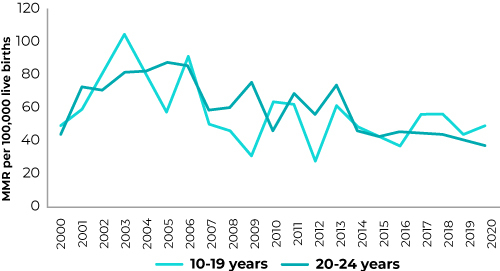
Historical series of the corrected cause-specific maternal mortality ratio (MMR) among adolescents (10-19 years old) and young adults (20-24 years old), state of Bahia, Brazil, 2000-2020

Between 2000 and 2020, in the state of Bahia, 3,136 maternal deaths were reported, of which 418 (13.3%) occurred in the 10-19 age group and 574 (18.3%) in the 20-24 age group. In the study period, the corrected CSMMR for adolescents was 59.7 deaths/100,000 LB, with a decrease from 104.7 (2003) to 50.1 (2020). Among young adults, the corrected CSMMR was 63.2 deaths/100,000 LB, with a decrease from 87.5 (2005) to 38.6 (2020) (Table 1 and Figure 1).

A statistically significant decreasing trend was observed in the trend lines for the CSMMR among adolescent women (APC = -2.2%; 95%CI -4.3;-0.01) and young adult women (APC = -2.9%; 95%CI -4.5;-1.4) during the study period (Table 1).

**Table 1:** Number of maternal deaths and annual percentage change of maternal mortality ratio, among adolescents (10 to 19 years old) and young adult women (20 to 24 years old), according to race/skin color, type and groups of causes and time of death, state of Bahia, Brazil, 2000-2020.

Variable	10 to 19 years old						20 to 24 years old					
	n	Total MMR^a^	APC^b^	_95%_ **CI** ^c^	p-value	Trend	N	Total MMR^a^	APC^b^	_95%_ **CI** ^c^	p-value	Trend
Total number of deaths	418	59.7^d^	-2.2	-4.3;-0.01	0.049	Decreasing	574	63.2^d^	-2.9	-4,5;-1.4	0.001	Decreasing
Race/skin color												
White	70	83.6	1.5	-2.3;5.5	0.413	Stability	79	66.4	-2.9	-18.1;15.1	0.722	Stability
Black	37	68.4	-16.5	-30.6;0.5	0.056	Stability	89	118.9	-31.5	-12.5;7.2	0.518	Stability
Mixed-race	259	34.4	2.8	0.3;5.4	0.031	Increasing	334	35.2	-0.1	-2.2;2.1	0.913	Stability
Direct causes	266	26.4	0.8	-1.1;2.7	0.393	Stability	360	27.7	-0.8	-2.2;0.6	0.254	Stability
Abortion	52	5.2	-4.7	-13.7;5.1	0.316	Stability	57	4.4	-0.5	-8.9;8.7	0.910	Stability
Hypertension	86	8.5	0.2	-7.7;8.9	0.951	Stability	112	8.6	-7.3	-13.7;-0.4	0.039	Decreasing
Hemorrhage	26	2.6	1.9	-9.7;15.0	0.746	Stability	61	4.7	-4.7	-15.1;7.0	0.397	Stability
Infections	40	4.0	9.5	-3.1;23.8	0.138	Stability	40	3.1	0.8	-7.1;9.5	0.837	Stability
Other obstetric complications	60	6.0	-0.08	-4.34;4.37	0.970	Stability	87	6.7	1.46	-1.67;4.70	0.345	Stability
Indirect causes	140	13.9	0.8	-3.9;5.6	0.740	Stability	192	14.8	-0.1	-4.4;4.4	0.969	Stability
Non-obstetric complications	140	13.9	0.8	-3.9;5.6	0.740	Stability	192	14.8	-0.1	-4.4;4.4	0.969	Stability
Unspecified cause	12	1.2	2.6	-9.2;16.0	0.662	Stability	22	1.7	21.2	11.2;32.0	0.000	Increasing
Unknown causes	12	1.2	2.6	-9.2;16.0	0.662	Stability	22	1.7	21.2	11.2;32.0	< 0.001	Increasing
Time of death												
During pregnancy, childbirth or abortion	146	14.5	2.9	-2.5;8.7	0.280	Stability	198	15.3	3.0	-6.5;13.5	0.532	Stability
Up to 42 days postpartum	133	13.2	11.0	5.1;17.3	0.001	Increasing	212	16.3	7.2	0.05;14.9	0.049	Increasing

a) Total MMR = total maternal mortality ratio; b) APC = annual percentage change; c) 95%CI = 95% confidence interval; d) Corrected total MMR

Information on ethnicity was not recorded in 12% of the cases, and timing of death (during pregnancy, delivery or postpartum) was not recorded in 25% of cases, with higher percentages of incompleteness in the first years of the time series.

As for race/skin color, among mixed-race adolescents, the CSMMR showed an increasing trend of 2.8% (95%CI 0.3;5.4) in the period (Table 1). The mean CSMMR was higher among Black adolescent and young adult mothers (132.9 and 194.1 maternal deaths/100,000 LB, respectively), when compared to white and mixed-race (Table 2).

**Table 2:** Comparison of means of the corrected total maternal mortality ratio, adolescents (10-19 years old) and young adults (20-24 years old), according to race/skin color, type of cause, and time of death, state of Bahia, Brazil, 2000-2020

Variable	10 to 19 years old			20 to 24 years old		
	Mean	Standard deviation	p-value	Mean	Standard deviation	p-value
Total MMR^a^	57.9	18.7		61	16.5	
Race/skin color^b^						
White	88.4	43.8	0.015	69.8	44.9	< 0.001
Black	132.9	176.8		194.1	235.4	
Mixed race	35.1	10.8		35.1	8.2	
Type of causes^b^						
Direct	26.6	7.0	< 0.001	27.6	5.4	< 0.001
Indirect	13.8	5.9		14.6	6.8	
Unspecified	1.2	1.2		1.9	1.8	
Time of death^c^						
During pregnancy, childbirth or abortion	14.6	7.00	0.906	15.2	8.7	0.439
Up to 42 days postpartum	14.3	8.3		17.4	9.6	

Total MMR: Total maternal mortality ratio; b) Analysis of variance (ANOVA); c) T-test for comparison of means.

In both age groups, the total CSMMR due to direct obstetric causes was predominant from hypertensive disorders (approximately 9.0 maternal deaths/100,000 LB), followed by other obstetric complications. Among adolescents, the third most frequent cause was abortion (5.2 maternal deaths/100,000 LB); while among young adults, it was hemorrhage (4.7 maternal deaths/100,000 LB). Regarding the groups of causes, group 2 (hypertensive disorders) showed a decreasing trend of 7.3% (95%CI -13.7;-0.4) among young adults (Table 1).

With regard to direct causes, the most frequent subcategories in both groups were: eclampsia; gestational hypertension with significant proteinuria; postpartum hemorrhage; and unspecified abortion. As for indirect causes, circulatory and respiratory system diseases stood out.

The period with the highest occurrence of maternal death among adolescents was during pregnancy, childbirth or due to abortion (14.5 maternal deaths/100,000 LB), while among young adults, it was the period up to 42 days postpartum (16.3 maternal deaths/100,000 LB). There was an increasing trend in maternal deaths occurring during this period (up to 42 days postpartum) in both age groups (11.0% among adolescents and 7.2% among young adults) (Table 1).

When comparing the CSMMR by means of ANOVA analysis, according to race/skin color and types of causes of death, according to age groups, it could be seen a significant difference between both groups (p-value < 0.05). In addition, the Tukey test showed that Black race/skin color differed significantly from other ethnicities, as well as the types of causes of death, in both age groups (Table 2).

Regarding the correlation analysis between the CSMMR and prenatal coverage, it could be seen an inverse and significant correlation between the highest frequency of prenatal care visits (> 6) and the lowest CSMMR in the adolescent group (r = -0.52; p-value = 0.017), as well as among young adults (r = -0.73; p-value < 0.001), although the correlation between lower frequency of prenatal care visits (≤ 6) and higher CSMMR was observed only in the young adult mothers group (r = 0.81; p-value < 0.001) (Table 3).

**Table 3:** Correlation coefficient between the maternal mortality ratio and the proportion of prenatal care visits, among adolescents (10-19 years old) and young adults (20-24 years old), state of Bahia, Brazil, 2000-2020

Variable	CSMMR^a^			
	10 to 19 years old		20 to 24 years old	
	r^b^	p-value	r^b^	p-value
Prenatal care coverage				
Did not have any prenatal care visits	0.34	0.138	0.42	0.057
≤ 6 prenatal care visits	0.32	0.151	0.81	< 0.001
> 6 prenatal care visits	-0.52	0.017	-0.73	< 0.001

a) CSMMR: Cause-specific maternal mortality ratio; b) r: Spearman’s correlation coefficient.

## DISCUSSION

In the state of Bahia, in the study period (2000 to 2020), the MMR among adolescent and young mothers still shows values below the parameters recommended by the WHO, despite the downward trend observed over two decades.^1^ ^),(^ ^3^ The historical series showed a decreasing trend in the CSMMR in both age groups, corroborating research findings worldwide, with a decrease in the global MMR (average annual rate of 2.1%) during the period.^1^ These findings point to the importance of investments at the national, state and municipal levels in the country, aimed at maternal and child health.^4^

In this investigation, deaths due to complications related to pregnancy, childbirth or postpartum, in both age groups, were higher among Black women. The results point to socioeconomic issues in the access to and maternal and child health care provided by the healthcare system.^20^ ^),(^ ^21^ Consistent with the results of other studies, these findings suggest the need to expand the range of strategies aimed at access and health care during the pregnancy and postpartum period, in order to reduce social inequalities among the most vulnerable population groups.^21^

This study, which uses aggregate data, contributes to corroborating research findings on the coefficient of maternal mortality among adolescents and young adults, regarding the insufficient number of prenatal care visits. The indicator used is the only one available on the SINASC database, contradicting the various criteria recommended by the WHO to assess the quality of prenatal care. This emphasizes the need for investments in primary health care and epidemiological surveillance sectors, aiming at adequate data collection and recording.^4^ ^),(^ ^10^

It is worth highlighting the important strategies implemented by the Ministry of Health including the National Pact for the Reduction of Maternal and Neonatal Mortality (2004); Companion Law (2005); Law No. 11.634 (Lei da Vinculação à Maternidade - 2007); Maternity Hospital Qualification Plan in the Northeast and Amazônia Legal (2009); Maternal-Child Health Service (2011); Guidelines for Maternal Care - cesarean section operation (2015); and National Guidelines for Assistance to Normal Childbirth (2016). This legislation has had a positive impact on investments in women’s health care, aiming to reduce maternal mortality.^4^

Regarding ethnic-racial aspects, studies have shown the occurrence of inequities in the profile of maternal mortality, with a higher frequency among Black, mixed-race and indigenous women.^22^ ^),(^ ^23^ The Birth in Brazil Survey, conducted with adolescents in postpartum period in 2011, observed a higher proportion of unfavorable conditions among Black women: lower adequacy of prenatal care, routine tests were not performed during pregnancy, low frequency of guidelines on pregnancy and childbirth, and greater need to seek health care during the labor process.^24^

As for the leading causes of maternal death, the results of this study indicated that the majority of maternal deaths among adolescents and young adults in the state of Bahia occur due to direct obstetric causes, especially hypertensive disorders, abortion, hemorrhage and puerperal infection. These results corroborate global research findings that show a majority of maternal deaths from these causes among young women.^25^ ^),(^ ^26^

This study shows an inverse and significant correlation between the highest number of prenatal care visits and the lowest CSMMR, in both age groups, confirming other findings.^3^ ^),(^ ^7^ ^)^ Research consensus shows that inadequacy of prenatal care is associated with a higher risk (i) of negative outcomes for maternal health, such as hypertensive disorders, hemorrhages and infection, (ii) for the newborn health such as prematurity and low birth weight, and (iii) of maternal and neonatal deaths.^10^ ^),(^ ^24^ ^),(^ ^27^ ^),(^ ^28^

The present investigation, based on two decades of data collected for the state of Bahia, agrees with studies conducted in different regions of Brazil that have shown a high frequency of inadequate prenatal care via the Brazilian National Health System (Sistema Único de Saúde - SUS), among young, Black women, without a partner, with low level of education and living in the poorest regions in the country (North and Northeast; Amazônia).^29^ ^),(^ ^30^ Given this reality, it is worth highlighting the need for sustainability of the strategies implemented by the SUS, at the national and regional levels, in order to ensure access and adequacy of health care.^27^ ^),(^ ^30^

There is a consensus in the world literature that maternal mortality reflects the socioeconomic status, in addition to the coverage and quality of health care provided to a population, representing one of the most sensitive indicators of human and social development.^2^ ^),(^ ^30^ However, despite advances in the quality of obstetric care, improvement in the coverage of Health Information Systems (HIS) and the implementation of the Maternal Mortality Committees (MMC) at the national level, maternal mortality during pregnancy and postpartum period remains high, making its reduction, a goal to be addressed by the SUS.^4^ ^)^ In this context, it is essential for healthcare professionals and managers to join efforts to contribute to the quality of obstetric care during prenatal care and childbirth.^28^ ^),(^ ^29^

Despite the results presented in this and other studies, it is worth highlighting some limitations related to the measurement of maternal mortality, using secondary data from national information systems. Studies have shown high rates of underreporting on the SIM, especially in the North and Northeast regions of Brazil, although, even in regions with excellent coverage of this record, the cause of maternal death is often not declared.^17^ ^),(^ ^18^

These findings indicate the need for investments in cause-of-death registration, taking into consideration the importance of these indicators for the development and implementation of policies that can make a difference in the health situation of the most vulnerable population groups.^4^ ^),(^ ^21^ In order to address this limitation, scholars recommend the use of correction factors in the calculation of maternal mortality indicators (MMR) based on data from official registry systems.^18^ Another limitation of the study is related to incomplete information in the fields of the Death Certificate (DC), regarding complications of pregnancy, childbirth and the postpartum period. These gaps may hinder estimates of indicators in this population group, reaffirming the need for investments in the epidemiological surveillance sector of the health system aimed at data capture and registration.The findings reinforce the importance of investments targeting the maternal and child population, especially in deprived regions and among the most vulnerable segments, in line with the SDG target, which proposes to reduce the global MMR to < 70 maternal deaths per 100,000 live births in the period between 2016 and 2030.^7^ ^),(^ ^30^
